# Carbon Ion Therapy Inhibits Esophageal Squamous Cell Carcinoma Metastasis by Upregulating STAT3 Through the JAK2/STAT3 Signaling Pathway

**DOI:** 10.3389/fpubh.2020.579705

**Published:** 2020-11-20

**Authors:** Hongtao Luo, Zhen Yang, Qiuning Zhang, Lihua Shao, Shihong Wei, Ruifeng Liu, Zheng Li, Yichao Geng, Chengcheng Li, Xiaohu Wang

**Affiliations:** ^1^Institute of Modern Physics, Chinese Academy of Sciences, Lanzhou, China; ^2^The First Clinical Medical College of Lanzhou University, Lanzhou, China; ^3^Lanzhou Heavy Ion Hospital, Lanzhou, China; ^4^The Basic Medical College of Lanzhou University, Lanzhou, China; ^5^Gansu Provincial Cancer Hospital, Lanzhou, China

**Keywords:** carbon ion beam, esophageal squamous cell carcinoma, apoptosis, metastasis, JAK2/STAT3 pathway

## Abstract

Radiation therapy is an important component of the comprehensive treatment of esophageal cancer. However, conventional radiation resistance is one of the main reasons for treatment failure. The superiority of heavy ion radiation in physics and biology has been increasingly highlighted in radiation therapy research. The Janus Kinase 2/Signal Transducer and Activator of Transcription 3 (JAK2/STAT3) pathway plays an important role in the occurrence, development and metastasis of esophageal squamous cell carcinoma (ESCC) and is related to the development of resistance to ionizing radiation in ESCC. Therefore, the aim of the present study was to investigate the relationship between carbon ion inhibition of the proliferation and metastasis of esophageal carcinoma cells and the JAK2/STAT3 signaling pathway. The results demonstrated that carbon ion beams significantly reduced cell viability and stimulated apoptosis in human ESCC cells in a dose-dependent manner. In addition, carbon ion beams induced G2/M phase cell cycle arrest in ESCC cells and inhibited tumor metastasis in a dose-dependent manner. Additionally, poorly differentiated KYSE150 cells were more sensitive to the same carbon ion beam dose than moderately differentiated ECA109 cells. Carbon ion beam exposure regulated the relative expression of metastasis-related molecules at the transcriptional and translational levels in ESCC cells. Carbon ion beams also regulated *CDH1* and *MMP2* downstream of the STAT3 pathway and inhibited ESCC cell metastasis, which activated the STAT3 signaling pathway. This study confirmed the inhibition of cell proliferation and the metastatic effect of carbon ion beam therapy in ESCC cells.

## Introduction

Esophageal cancer is the eighth most prevalent cancer worldwide and is the sixth leading cause of tumor-related death (1). Radiation therapy is one of the mainstays of treatment for esophageal cancer, but in China, 95% of esophageal cancers are esophageal squamous cell carcinomas (ESCCs) (2). ESCC is relatively resistant to traditional X-ray radiotherapy and has poor clinical outcomes, and the main causes of failure are local uncontrolled growth or/and recurrence. Studies have shown that when high linear energy transfer (LET) ray–carbon ion irradiation is applied, cell damage primarily involves DNA double-strand breaks (DSBs). Clinical studies have shown that carbon ion radiotherapy has a good clinical effect on refractory or recurrent tumors treated with conventional photon radiotherapy. At present, not many mechanisms by which carbon ion radiation induces tumor cell apoptosis and inhibits metastasis have been established. Signal transducer and activator of transcription 3 (*STAT3*) was recognized as an oncogene for its role in the malignant transformation of cells and tumorigenesis (3, 4). STAT3 protein is activated by tyrosine phosphorylation at residue 705, and p-STAT3 (Tyr705) is able to upregulate the transcription of genes involved in cell proliferation, apoptosis, angiogenesis, invasion and metastasis. Carbon ion beams can alter the biological characteristics of tumor cells. However, the mechanisms by which carbon ion beams function remain unclear, especially in terms of how they regulate the JAK2/STAT3 signaling pathway.

## Materials and Methods

### Cell Culture and Irradiation Conditions

Human ESCC cells (ECA109, KYSE150) were obtained from the Shanghai Genechem Co., Ltd. The cells were maintained in RPMI1640 medium supplemented with 10% (v/v) fetal bovine serum (FBS), 100 U/mL penicillin and 100 mg/mL streptomycin (Life Technologies) in a humidified atmosphere of 5% (v/v) CO_2_ and 95% (v/v) air at 37°C. The medium was changed every other day, and cells in the logarithmic growth phase (1 × 10^7^ cells were harvested at 70% confluence) were used for subsequent experiments. Heavy ions were obtained from the Cooling Storage Ring Project of the Heavy Ion Research Facility in Lanzhou (HIRFL-CSR) (Ray parameters: energy of 100 MeV, dose rate of 1 Gy/min, broadened Bragg peak of 5 mm, radiation field of 5 × 5 cm). For cell irradiation, heavy ions were obtained from the carbon ion (^12^C^6+^) beam of the Deep Therapy Terminal, Institute of Modern Physics, Chinese Academy of Sciences (HIRFL-CSR). Irradiation doses were 0, 1, 2, and 4 Gy. This experiment was repeated three times.

### Colony Formation Assay

Cells in the logarithmic growth phase were detached with 0.25% trypsin and then triturated into single cells and centrifuged. The number of cells was counted with a counting board. After they were resuspended in culture medium containing 10% FBS, ECA109 and KYSE150 cells were inoculated separately into a 6-well plate after, as follows: 0 Gy group, 200 cells/well; 1 Gy group, 1,000 cells/well; 2 Gy group, 2,000 cells/well; and 4 Gy group, 4,000 cells/well. Three replicate wells were used for each group. After an overnight inoculation, the cells were exposed to 0, 1, 2, or 4 Gy carbon ion rays and cultured in a cell incubator for 12 days. When the cell colonies were visible to the naked eye, approximately 50 cells were counted under the microscope, and the experiment was terminated. Next, the cells were fixed in 4% polyformaldehyde (500 μL) for 15 min, stained with a crystal violet solution (500 μL) for 15 min and observed by microscopy. The plating efficiency (PE) = colony number/inoculation number × 100%, and the survival fraction (SF) = colony rate in the experimental group/colony rate in the control group × 100%. The cell dose survival curve was generated using the formula S = 1-(1-e–KD)N and GraphPad Prism 6 software. This experiment was repeated three times.

### Cell Counting Kit-8 (CCK-8) Assay

ECA109 and KYSE150 cells cultured in individual wells were detached with trypsin and resuspended in a small volume of culture medium, after which the cells were counted. Next, 100 μL (~10,000 cells) of the cell suspension was added to each well of a 96-well plate. Six replicate wells were used for each group, and the culture plate was placed in an incubator for a 24-h pre-culture (37°C, 5% CO_2_) to allow the cells to adhere. To avoid the influence of water volatilization on cell growth and the experimental results, 200 μL PBS was added to each well of the culture plate. When the cells reached 50–60% confluence, they were exposed to 0, 1, 2, or 4 Gy carbon ion rays. Cell proliferation was detected at 24, 48, and 72 h after irradiation. Cells in each well were then incubated with 10 μL CCK-8 solution for 2 h. The optical density (OD) value of each well was detected by a microplate reader (450 nm). The cell survival rate = (OD value in each irradiation group-OD value in each blank group)/(OD value in the non-irradiation group-OD value in each blank group) × 100%. This experiment was repeated three times.

### Cell Cycle and Apoptosis Analysis

Cells were seeded into Φ60 plates for 24 h and exposed to 0, 1, 2, or 4 Gy carbon ion radiation when they reached 50–60% confluence. Next, the cells were prepared by trypsin digestion after which 1 × 10^3^ cells/ml were collected for the detection of cell cycle distribution. The cell cycle distribution was detected at 24 h. Cells were then labeled with propidium iodide (PI), and 1 × 10^4^ cells were collected by flow cytometry for cell cycle analysis (BD Biosciences, San Jose, CA, USA). ECA109 and KYSE150 cells were irradiated with 0, 1, 2, or 4 Gy of carbon ion beams and then cultured for 24 h. The cells were detached with EDTA-free trypsin and then washed with pre-cooled PBS. The dead cells floating in the medium were also collected. The cells were suspended in 100 μL of 1 × binding buffer and prepared as a single cell suspension with a density of 1 × 10^6^ cells/ml. After treatment, the cells were harvested with trypsin and washed twice with cold PBS. The cells were then stained with Annexin V for 10 min in the dark and then stained with PI for 5 min. Annexin V-binding buffer was then added to the mixture before fluorescence was measured on a FACSCalibur flow cytometer (BD Biosciences; Baltimore, MD, USA). In all, 2 × 10^4^ cells were collected by flow cytometry for apoptosis analysis. The data were analyzed using Flow Jo software.

### Wound-Healing Assay

Wound-healing assays were performed according to a routine protocol, as follows: 3 × 10^5^ ECA109 and KYSE150 cells were seeded in each well of a 6-well plate in RPMI 1640 medium with 10% FBS and cultured at 37°C with 5% CO_2_ for 24 h until a monolayer was formed. A scratch wound was generated at the bottom of the plate using a sterile 10-μL pipette tip. After three washes in PBS, the cells were irradiated with 0, 1, 2, or 4 Gy carbon ion beams and then cultured for 24 h, which allowed examination of cell migration in the absence of cell growth. Wound closure was measured in three random fields in each well using an inverted microscope and was compared with that of the control group. Each group was assayed in triplicate.

### Transwell Assay

A Transwell assay to determine cell invasion was performed according to the following protocol. Polycarbonate membranes with an 8-μm pore (Corning, USA) were placed in 24-well Transwell plates (Corning, USA). Briefly, the polycarbonate filters were coated with Matrigel at a concentration of 1 μg/mL and placed in a modified Boyden chamber. The irradiated cells were prepared by trypsin digestion after which cells (3 × 10^4^) resuspended in RPMI 1640 medium containing 1% (v/v) FBS were added to the top chamber. Culture medium containing 5% (v/v) FBS was then added to the bottom chamber. Cell motility/migration was measured as the number of cells that migrated from a defined area on the uncoated microfilter through micropores over a given time (24 h). The cells that migrated to the lower surface of the membrane were fixed in methanol for 10 min and stained with 0.5% (w/v) crystal violet for 30 min. All experiments were performed in triplicate, and a minimum of five fields per filter was counted.

### RNA Isolation and Quantification

Total RNA was extracted from cells using TRIzol (Takara Co., Ltd., Dalian, China), after which the concentration and purity of the RNA were determined. For the detection of *STAT3, MMP2* and *CDH1* mRNA, reverse transcription primers (synthesized by Suzhou GENEWIZ Co., Ltd., Suzhou, China) and a reverse transcription kit (Takara Co., Ltd., Dalian, China) were used to reverse-transcribe cDNA. SYBR Green Real-Time PCR Master Mix was used for real-time polymerase chain reaction (PCR) assays, which were performed in an ABI 7500 real-time PCR system. The following primer sequences were used for real-time PCR: *STAT3*-F: 5′-ACCAGCAGTATAGCCGCTTC-3′; *STAT3*-R: 5′-GCCACAATCCGGGCAATCT-3′; *MMP2*-F: 5′-GATGGCATCGCTCAGATCCG-3′; *MMP2*-R: 5′-TCAG GCCAGAATGTGGCCAC-3′; *CDH1*-F: 5′-AGAGGTGGGTGACTACAAA-3′; *CDH1*-R: 5′-TCTCCTCCGAAGAAACAG-3′;JAK2-F:5′-GCTCAGTGGCGGCATGAT-3′;5′-CACTGCCATCCCAAGACATTC-3′; *ACTB*-F: 5′-CGGGAAATCGTGCGTGAC-3′; *ACTB*-R: 5′-GAAGGAAGGCTGGAAGAGT-3′. *ACTB* was used as an internal control, and 2^−ΔΔ*Ct*^ was used to calculate gene expression. This experiment was repeated three times.

### Western Blot Analysis

Cells were washed with ice-cold PBS and total proteins were extracted in lysis buffer (Solarbio, Beijing). The protein concentrations were determined by a Bradford assay. The cell lysates were mixed with 5x sodium dodecyl sulfate (SDS) sample buffer, boiled for 5 min, and then separated by 10% (w/v) SDS-PAGE. After electrophoresis, the proteins were transferred to polyvinylidene difluoride (PVDF) membranes, which were blocked in 5% (w/v) non-fat dry milk or BSA for 30 min. Membranes were rinsed and incubated with the following specific antibodies against MMP2 (mouse monoclonal, 1:2,000) (GTX27033, GeneTex, USA), ACTB (mouse monoclonal, 1:2,000) (GTX26272, GeneTex, USA), JAK2 (YT2429, rabbit, Immunoway, 1:1000), STAT3 (rabbit polyclonal, 1:1,000) (cat. no. 9139T, CST, USA), Phospho-Stat3 (Tyr705) (rabbit polyclonal, 1:1,000) (cat. no. 9145, CST, USA), and CDH1 (mouse monoclonal, 1:2,000) (GTX629691, GeneTex, USA) in Tris-buffered saline containing 5% (w/v) non-fat dry milk and Tween-20 (0.1% (v/v)) (TBST) overnight at 4°C. After washing, the signals were detected with a horseradish peroxidase-conjugated secondary antibody (1:1,000, cat. no. ZDR-5307, ZSGB. BIO, China) for 1 h and were washed three times in TBST. Finally, immunopositive bands were visualized using an enhanced chemiluminescence (ECL) system (Amersham Pharmacia Biotech) and were exposed using Image Lab 3.0 (Bio-Rad, USA).

### Statistical Analyses

The statistical significance between groups was determined using either the ANOVA test followed by the Bonferroni post-test when applicable or the Mann-Whitney *U*-test. Data are presented as the mean ± SD. All experiments were performed at least three times using new frozen batches of cells to maintain independence among replicates. Analysis was performed and graphs were generated using GraphPad Prism software 5.0.

## Results

### Carbon Ion Beams Inhibit Cell Colony Formation and Cell Proliferation

**Figure 1:**
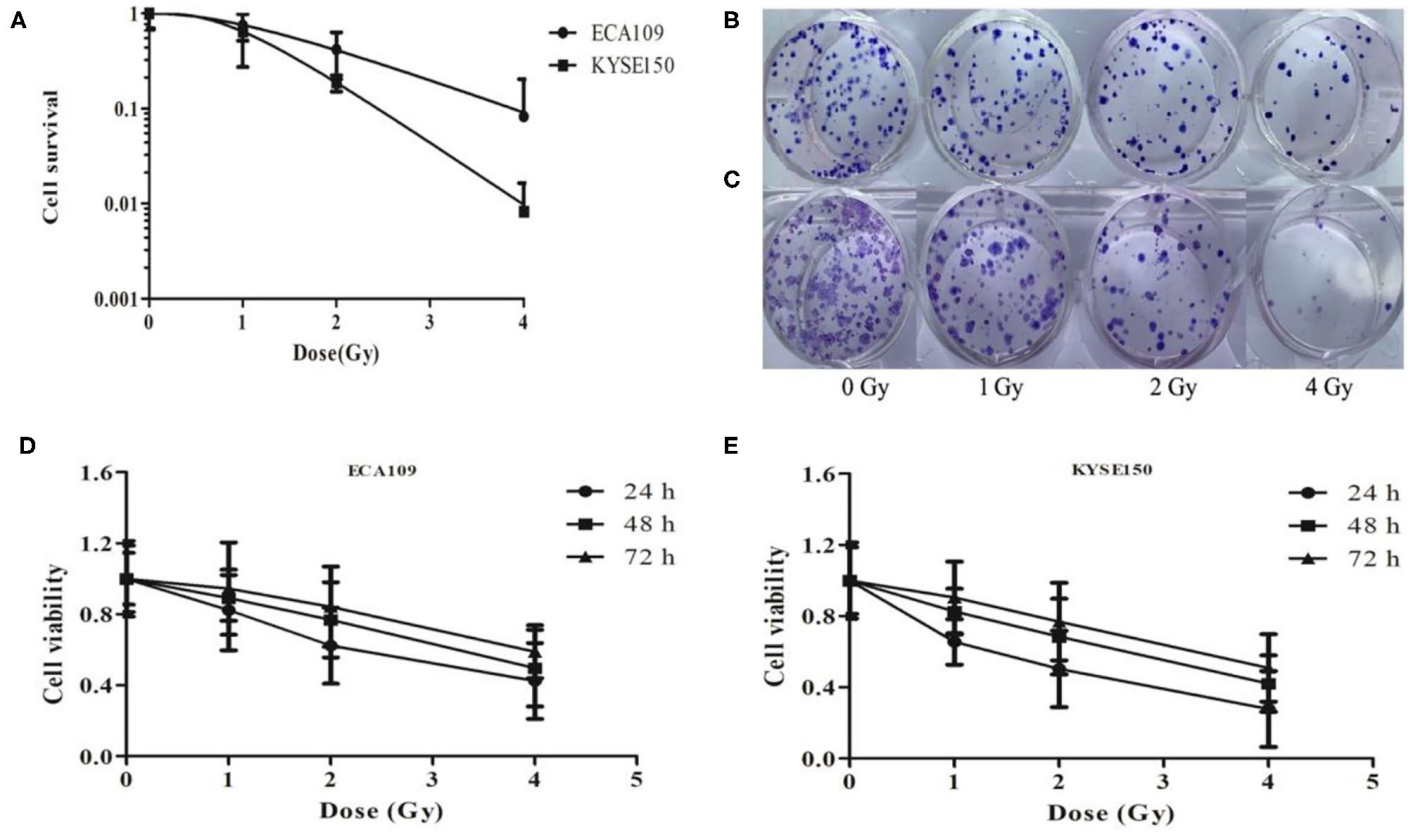
Carbon ion beam therapy inhibits ESCC cell proliferation. **(A)** Clonogenic survival curves of ECA109 and KYSE150 carcinoma cell lines after carbon ion therapy. **(B,C)** Colony formation of ECA109 and KYSE150 cells. **(D,E)** Carbon ions inhibit the proliferation of ECA109 and KYSE150 cells.

We analyzed the radiosensitivity of two differentiated esophageal cancer cell lines (ECA109 and KYSE150) to carbon ions. The results confirmed that colony formation ability was significantly lower in cells treated with 1, 2, and 4 Gy compared with cells treated with 0 Gy (Figure 1A). A positive correlation was observed between the colony formation inhibition rate and the carbon ion irradiation dose. The survival of both cell lines decreased after treatment with 1 Gy irradiation compared with treatment with 0 Gy, but the difference was not significant (Figures 1B,C); however, after 2 and 4 Gy irradiation, cell survival decreased significantly (*p* = 0.031). Taken together, these data clearly demonstrate that carbon ions can effectively inhibit ESCC cell proliferation.

The CCK-8 assay results revealed different growth changes after 24, 48, and 72 h. After 1 Gy irradiation, ECA109 and KYSE150 cell proliferation was significantly inhibited at 24 h, and this inhibition increased in a dose-dependent manner; ECA109 cell proliferation inhibition showed a decreasing trend at different times after irradiation, but no significant differences were found among the various time points (Figures 1D,E). Compared with ECA109 cells, KYSE150 cells were more sensitive to heavy ion irradiation, and KYSE150 cell proliferation was significantly inhibited 24 h after 1 Gy irradiation (*p* = 0.042). However, cell proliferation inhibition was not significant at 48 and 72 h. In contrast, KYSE150 cell proliferation was significantly inhibited after 2 and 4 Gy irradiation.

### Carbon Ion Beams Induce Apoptosis in Human ESCC Cells

**Figure 2:**
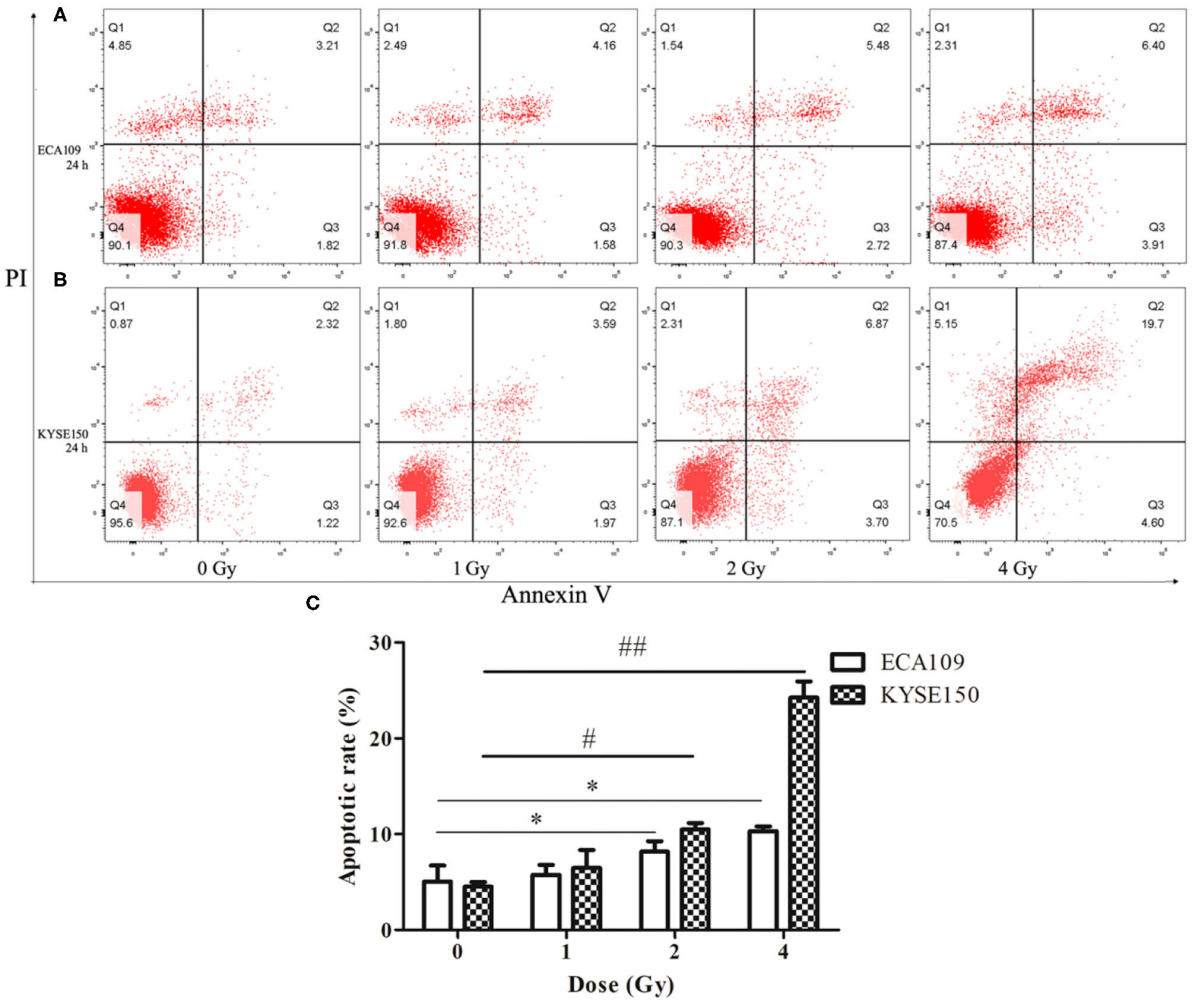
Carbon ion-induced apoptosis. **(A,B)** Apoptosis of ECA109 and KYSE150 cells 24 h after irradiation. **(C)** Carbon ion irradiation promotes apoptosis of ECA109 and KYSE150 cells. (*,#*p* < 0.05, ##*p* < 0.01).

Compared with the 0 Gy group, the 2 and 4 Gy groups exhibited enhanced inhibition of cell viability in the tested cell lines. At 24 h after irradiation, more dead ECA109 cells were observed (Figure 2A) than after 0 Gy; the apoptosis rate was significantly higher after 1, 2 and 4 Gy irradiation, and the apoptosis rates were highest in KYSE150 cells after 2 and 4 Gy irradiation (*p* = 0.048, *p* = 0.027, Figure 2B). The number of apoptotic cells was positively correlated with the irradiation dose. The apoptosis rates of ECA109 cells and KYSE150 cells were different after the same dose of carbon ion irradiation, as KYSE150 cells were more sensitive to carbon ion beams (Figure 2C).

### Carbon Ion Beams Induce G2/M Cell Cycle Arrest in Human ESCC Cells

**Figure 3:**
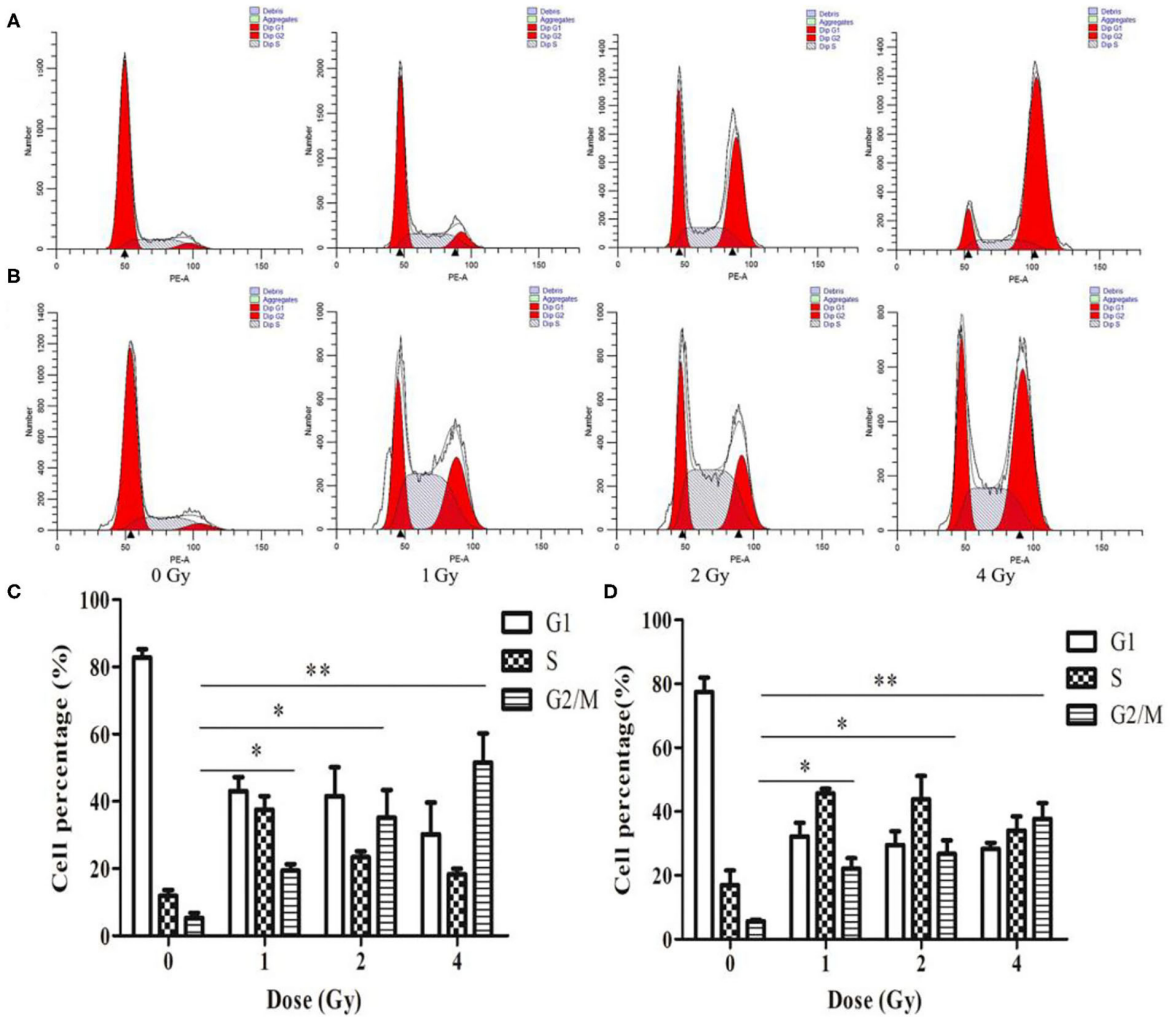
Changes in cell cycle arrest 24 h after irradiation of ECA109 and KYSE150 cells with carbon ion rays. **(A,B)** Cell cycle distribution of ECA109 and KYSE150 cells 24 h after irradiation. **(C,D)** Inhibition of cell cycle arrest in ECA109 and KYSE150 cells by carbon ions. *p < 0.05, **p < 0.01.

The cell cycle involves a series of cellular events that lead to cell division and eventually proliferation. After 0, 1, 2 and 4 Gy irradiation, the number of ECA109 and KYSE150 cells in G2/M phase arrest increased in a dose-dependent manner within 24 h compared with after 0 Gy irradiation (Figures 3A,B). Twenty-four hours after carbon ion irradiation, significantly more KYSE150 cells were in S phase arrest after 1, 2 and 4 Gy irradiation, whereas the number of cells in S phase arrest was decreased after 4 Gy (Figure 3B). S phase arrest increased after 1 Gy irradiation, but G2/M phase arrest was not significantly different (*p* = 0.69), while G2/M phase arrest was significant after 2 and 4 Gy irradiation (*p* = 0.025, *p* = 0.013, Figure 3C). G2/M arrest also increased in a dose-dependent manner, and the increase was most significant after 4 Gy (*p* = 0.010, Figure 3D).

### Carbon Ion Beams Inhibit Migration and Invasiveness of ECA109 and KYSE150 Cells

**Figure 4:**
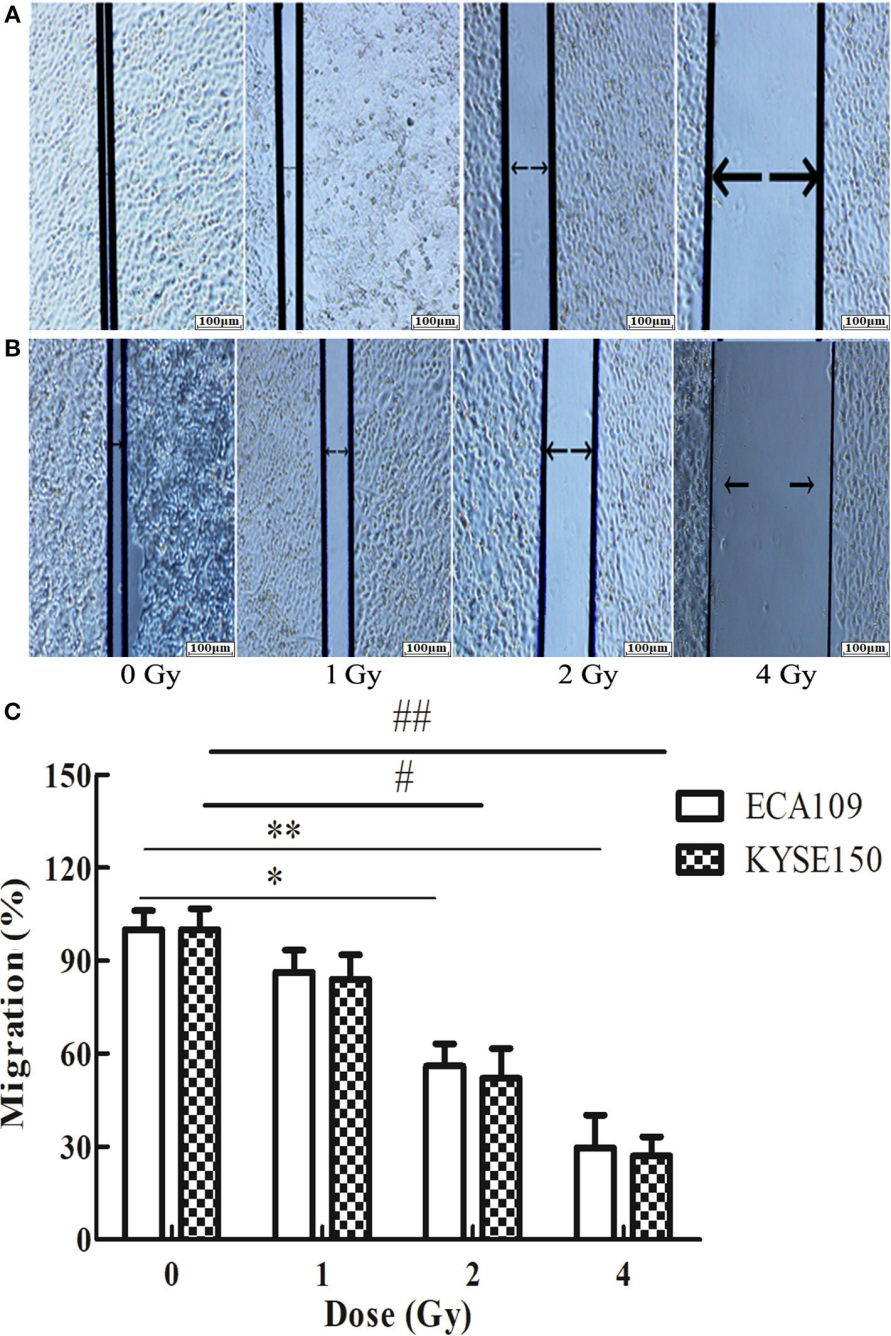
The effect of carbon ion beam irradiation on ECA109 and KYSE150 cell migration as shown by a wound-healing assay. **(A,B)** Scratch area of ECA109 and KYSE150 cells 24 h after irradiation. **(C)** Inhibition of ECA109 and KYSE150 cell migration by carbon ions (*,#*p* < 0.05, **,##*p* < 0.01).

**Figure 5:**
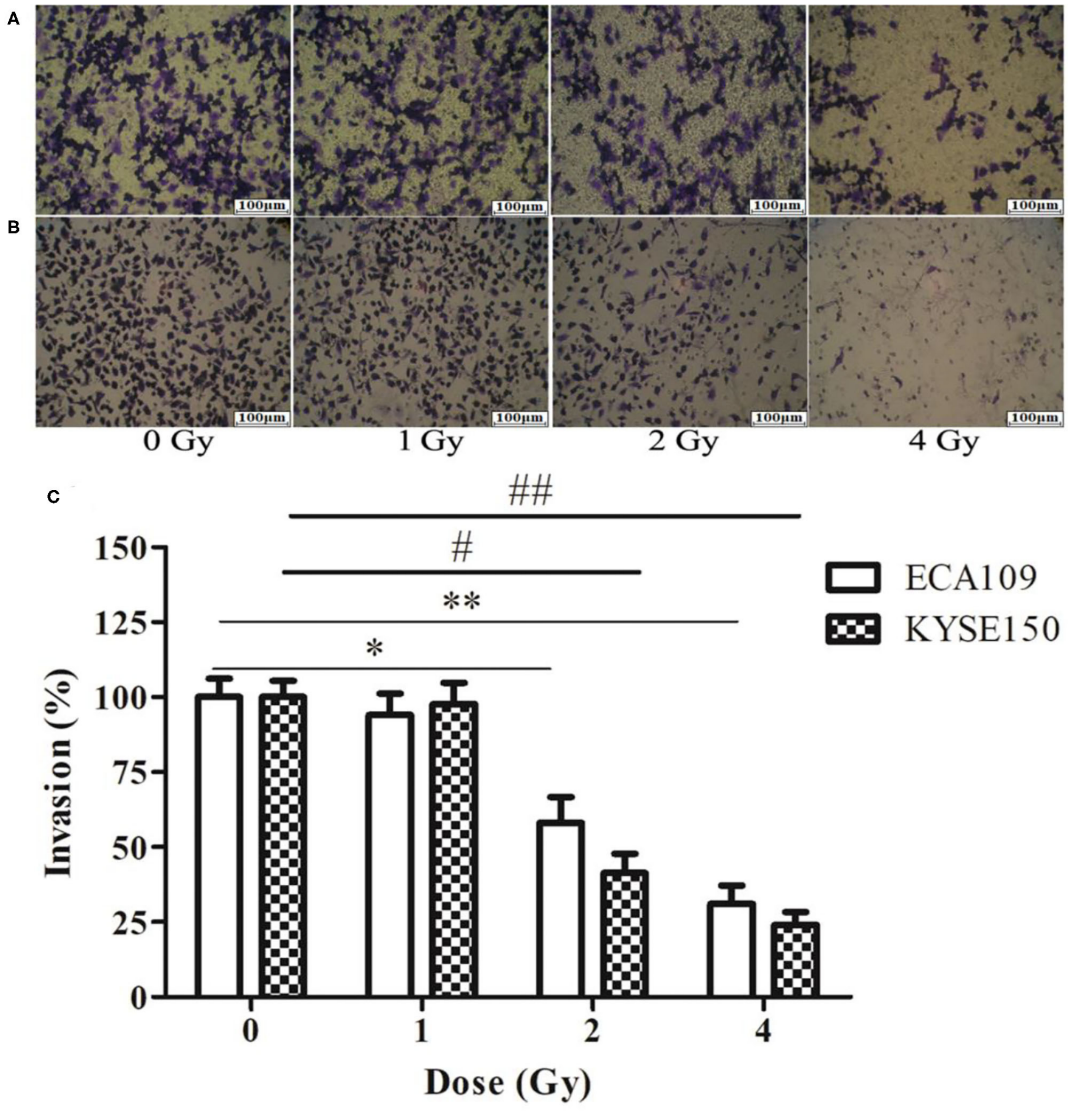
The effect of carbon ion beam irradiation on ECA109 and KYSE150 cell invasiveness by Transwell assay. **(A)** Number of invasive ECA109 cells after irradiation. **(B)** Number of invasive KYSE150 cells after irradiation. **(C)** Inhibition of ECA109 and KYSE150 cell invasion by carbon ions (*,#*p* < 0.05, **,##*p* < 0.01).

The wound-healing assay showed that the relative migration and invasiveness of ECA109 cells was significantly lower 24 h after 2 and 4 Gy irradiation than 24 h after 0 Gy irradiation (*p* = 0.045, *p* = 0.028, Figure 4A). A positive correlation was observed between the number of KYSE150 cells that had migrated and invaded and the irradiation dose (Figures 4B,C). However, migration and invasion in the 1 Gy group were not significantly different (*p* = 0.960, Figures 5A,B). At 24 h after irradiation, the number of migrating and invading KYSE150 cells was significantly lower (*p* = 0.031, *p* = 0.010, Figure 5C) after 2 and 4 Gy irradiation than after 0 Gy irradiation but was not significantly different after irradiation with 1 Gy (*p* = 1.25).

### Carbon Ion Beams Regulate Metastasis-Related Expression of Proteins in the JAK2/STAT3 Signaling Pathway

**Figure 6:**
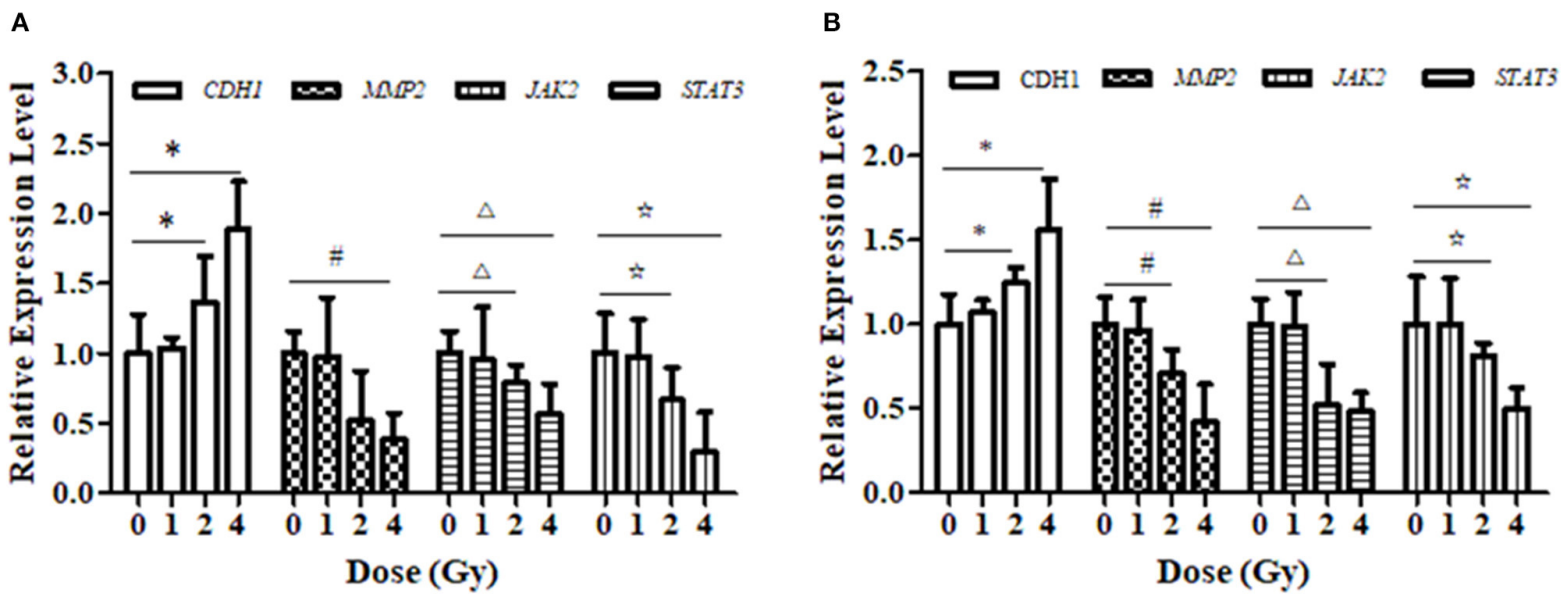
Effect of carbon ion irradiation on the transcription level of *STAT3*-related molecules in ECA109 and KYSE150 cells. **(A)** The relative expression of genes related to carbon ion regulation at 24 h after carbon ion irradiation was measured by qRT-PCR in ECA109 cell. **(B)** The relative expression of genes related to carbon ion regulation at 24 h after carbon ion irradiation was measured by qRT-PCR in KYSE150 cell. (*,^#^,^Δ^,^⋆^, *p* < 0.05).

The relative expression levels of *CDH1, JAK2*, and *STAT3* mRNA in ECA109 cells were significantly different (*p* = 0.027, *p* = 0.034 and *p* = 0.015, Figure 6A) after 2 and 4 Gy carbon ion irradiation compared with those in the 0 Gy group. *MMP2* mRNA was differentially expressed after 4 Gy and was not significantly expressed after 1 Gy irradiation (*p* = 0.081). At 24 h, *CDH1* expression was upregulated and *MMP2, JAK2* and *STAT3* mRNA expression was downregulated after 2 and 4 Gy irradiation in KYSE150 cells, and these differences were significant (*p* = 0.017, *p* = 0.042, and *p* = 0.29, Figure 6B); in contrast, the expression levels were not significantly altered after 1 Gy irradiation (*p* = 0.076, *p* = 0.052, and *p* = 0.108).

**Figure 7:**
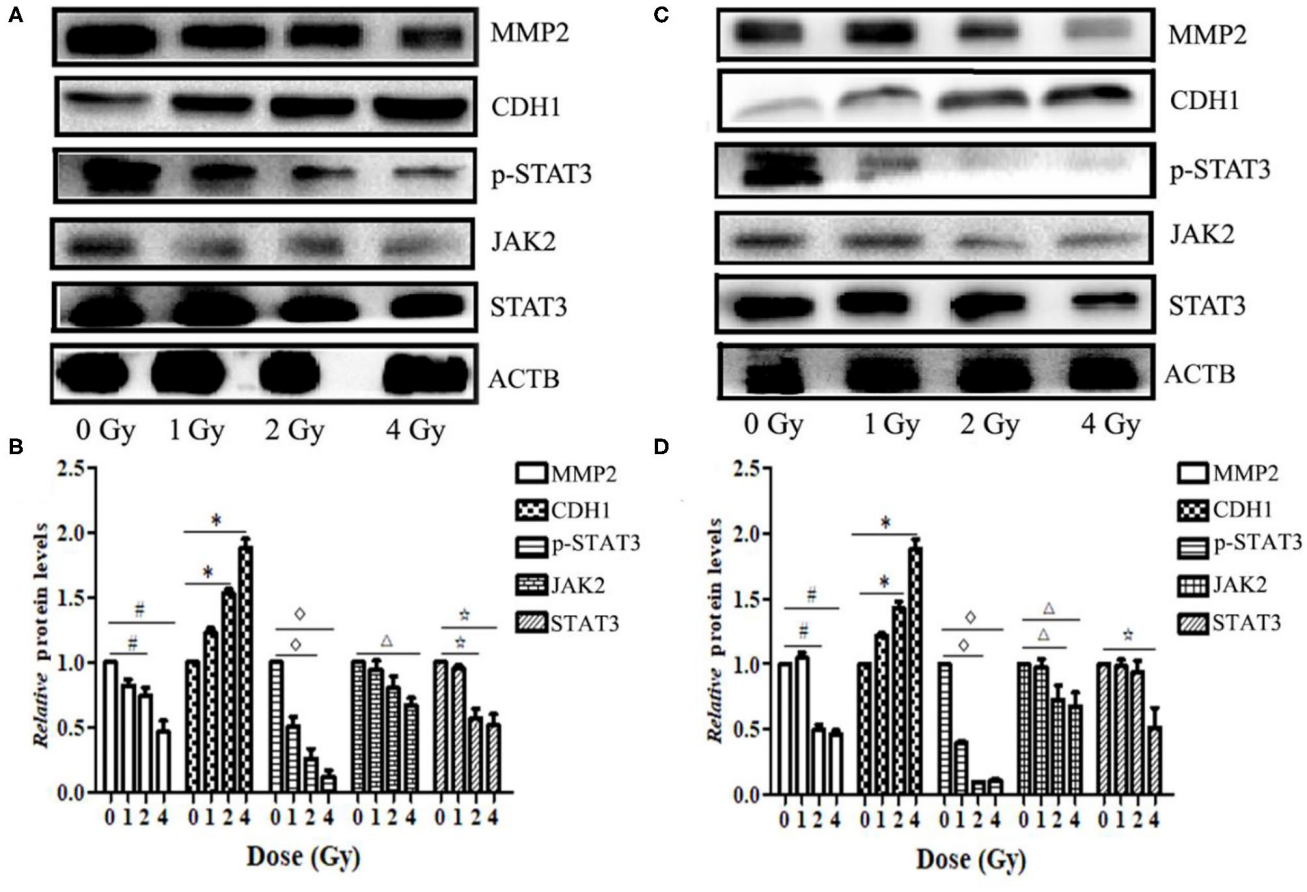
Carbon ion beams inhibit ECA109 and KYSE150 cell migration via regulation of JAK2/STAT3 signaling. **(A,C)** Western blotting analysis of protein expression changes in ECA109 and KYSE150 cells 24 h after irradiation. **(B,D)** Effect of carbon ion irradiation on relative protein expression in ECA109 and KYSE150 cells (*,^#^,^Δ^, ^⋆^, *p* < 0.05).

Western blotting results showed that the expression levels of MMP2 and STAT3 proteins in ECA109 and KYSE150 cells were reduced at 24 h after different doses of carbon ion beam irradiation (Figures 7A,B), and the expression levels of both proteins were lower after 2 and 4 Gy irradiation than after 0 Gy irradiation (*p* = 0.136 and *p* = 0.265, *p*>0.05). Carbon ion irradiation of 2 and 4 Gy significantly reduced the expression of JAK2 in KYSE150 cells, while in ECA109 cells, the expression of JAK2 was significantly downregulated after 4 Gy irradiation (Figures 7C,D). The expression level of CDH1 (E-cadherin) protein was increased, with the highest protein expression observed after 4 Gy irradiation (Figure 7C). No significant difference was seen in the expression levels of these three proteins after 1 Gy irradiation. In KYSE150 cells, the expression levels of MMP2, STAT3 and p-STAT3(Tyr705) were significantly altered after 4 Gy irradiation (Figure 7D).

## Discussion

ESCC is a common cancer type worldwide and accounts for an estimated 90% of the most malignant esophageal tumors (5). The treatment of ESCC is generally limited to surgical resection, chemotherapy and radiotherapy approaches, but the results are often largely unsatisfactory (6). Surgery is the treatment of choice for EC, but 80% of patients with EC are no longer candidates for radical surgery at the time of diagnosis (7), and thus, radiotherapy is the primary treatment for patients with advanced EC (8). Recent studies have indicated that ESCC progression occurs as a result of synergy among multiple genes (9). In recent years, with the continuous development of radiotherapy equipment and technology, carbon ion beams with high-energy transmission of linear density rays not only have the physical advantages of Bragg peak dose distributions compared with photons, but they also have greater radiobiological effects. The relative biological effectiveness (RBE) value of the Bragg peak area (tumor target area) is high, and clustered DNA damage is predominant in damaged and dying cancer cells. This damage is not dependent on the cell cycle or on the oxygen enhancement ratio where the cells are located and cannot easily be repaired (10, 11). Inhibition of the JAK2/STAT3 signal transduction pathway is expected to be an effective way to inhibit tumor growth and promote tumor cell apoptosis. It has been shown that STAT3 can be used as a marker of ESCC cell proliferation (12, 13). According to the results of this study, after carbon ion irradiation was applied to moderately differentiated cells (ECA109) and poorly differentiated cells (KYSE150), proliferation was inhibited after 2 and 4 Gy irradiation, and the primary effect of ionizing radiation was to induce DNA molecule breakage in the cells, thereby inhibiting cell proliferation. Cells subjected to ionizing radiation, which initiates protein repair at DSBs (14, 15), demonstrate a clear time-dependent linear relationship with the radiation dose (16, 17). A small effect of oxygen is also observed during heavy ion beam irradiation with a LET of more than 200 keV/μm (18).

In this study, we found that the cell cycle in two ESCC cell lines with different degrees of differentiation was arrested at the G2/M phase after carbon ion irradiation and that the number of cells in cell cycle arrest was positively correlated with the irradiation dose. Furthermore, KYSE150 cells were more sensitive than ECA109 cells to carbon ion radiation. Over time, the radiation effect caused by the release of powerful energy from the heavy ion beam at the end of the range causes the double-stranded DNA of the target gene to become irreparably damaged (19), which leads to apoptosis. In recent years, *in vitro* studies have shown that heavy ion beam (^12^C^6+^) irradiation of lung cancer cells induces G2/M arrest, increases the rate of apoptosis and inhibits tumor cell invasion and metastasis (20, 21). The results showed that heavy ion irradiation could induce G2/M phase arrest and that heavy ion beams caused DNA DSBs, which cause irreparable lethal damage and effects on downstream signaling (22–24); this in turn initiates apoptotic mechanisms (25). In our study, a correlation was found between the migration rate of both cell lines and the irradiation dose, and poorly differentiated KYSE150 cells were more sensitive to carbon ion radiation. The effect of carbon ion rays on cell mobility was not correlated with the length of time after irradiation. Heavy ion beams can exert anti-tumor effects by upregulating the pro-apoptotic gene *BAX* and inhibiting the anti-apoptotic gene *BCL2*, which inhibits *MMP2* and *MMP9* expression and angiogenesis in tumors (26). We discovered that 24 h after carbon ion irradiation of two ESCC cell lines with different degrees of differentiation, *CDH1* expression was upregulated, while that of *MMP2* and *STAT3* was downregulated at the transcriptional level in ECA109 cells; these changes consequently inhibited the metastasis of tumor cells. The upregulated expression of *CDH1* may be related to epithelial-mesenchymal transition (EMT), and we suspect that the transition between *CDH2* and *CDH1* expression occurs after carbon ion irradiation. The core function of *CDH1* is undoubtedly determined by multiple forms of regulation, such as promoter methylation, histone methylation, transcriptional inhibition, and post-transcriptional modification-mediated endocytosis (27–29). Tumor metastasis may be the result of the joint action of these molecules, and specific experiments have confirmed this in follow-up studies. The relative expression of *CDH1, MMP2* and *STAT3* mRNA in poorly differentiated KYSE150 cells was correlated with the irradiation dose. The expression levels of these proteins are possibly altered through downregulation of the relative MMP2, p-STAT3(Tyr705) and STAT3 expression at the transcriptional and translational levels, upregulation of *CDH1* expression, and co-regulation of EC cell metastasis by carbon ion beams. It has been shown that phosphorylation of STAT3 can increase cell-cell contact, and thus, STAT3 may be an essential gene for the migration and invasiveness of tumor cells (30–32).

It has been shown that STAT3 protein binds to a high-affinity binding site in the promoter region of the *MMP2* gene, which upregulates *MMP2* expression. Inhibition of STAT3 inhibits its target gene *MMP2* and inhibits tumor cell invasion (33). In malignant tumors, E-cadherin, an epithelial marker, is downregulated, and vimentin, an interstitial marker, is upregulated; moreover, cell adhesion is decreased, migration is enhanced, and tumor cells are more susceptible to invasion and metastasis (34). In various human malignancies, aberrant activation of the STAT3 signaling pathway is closely associated with tumor EMT, invasion and metastasis (35). Many studies have shown that inducing the inactivation of JAK2 can inhibit STAT3 activation and can thus inhibit STAT3 signaling, which then regulates apoptosis of cancer cells. The elevated expression of p-STAT3(Tyr705) is the result of sustained activation of the STAT3 signaling pathway, which affects the energy needed for cell proliferation and invasion (36). In addition, many studies have shown that *STAT3* over-expression significantly decreases the expression of *CDH1* and that inhibition of *STAT3* expression significantly increases *CDH1* expression (37, 38). Activation of STAT3 signaling leads to the decreased expression of *CDH1*, which encodes E-cadherin, an epithelial marker in ESCC cells that promotes cell invasion and metastasis (39). In melanoma cells, the STAT3 signaling pathway can promote *MMP2* expression, while inhibition of phosphorylated STAT3 expression can significantly inhibit *MMP2* expression *in vivo*, which inhibits tumor cell growth and invasion (40).

In conclusion, our findings indicate that carbon ions inhibit the sustained activation of STAT3 through the JAK2/STAT3 pathway, which inhibits the migration and invasiveness of esophageal cancer cells. We also found that high doses of carbon ions prolonged G2/M cell cycle arrest, promoted apoptosis and significantly inhibited cell proliferation.

## Data Availability Statement

The raw data supporting the conclusions of this article will be made available by the authors, without undue reservation.

## Consent for Publication

Written informed consent for publication was obtained from each participant.

## Author Contributions

HL, XW, and QZ: conception and design. XW, HL, and SW: administrative support. HL, ZY, LS, SW, RL, YG, and CL: experimental testing. HL, QZ, ZY, ZL, and SW: data analysis and interpretation. All authors contributed to the article and approved the submitted version.

## Conflict of Interest

The authors declare that the research was conducted in the absence of any commercial or financial relationships that could be construed as a potential conflict of interest.
